# Protecting kidney function: from mechanisms to therapeutic targets and traditional Chinese medicine

**DOI:** 10.1080/0886022X.2025.2539936

**Published:** 2025-07-31

**Authors:** Yilin Meng, Lu Sui, Ligeer Che, Zihang Jin, Yutong Ma, Li Sun

**Affiliations:** Department of Nephrology, The First Hospital of China Medical University, Shenyang, People’s Republic of China

**Keywords:** Chronic kidney disease, renal fibrosis, mechanism, therapy, traditional Chinese medicine

## Abstract

Chronic kidney disease (CKD) has been a significant public health concern globally with its prevalence increasing yearly. Renal fibrosis is the ultimate outcome of CKD progression to end-stage renal disease. However, effective treatment for renal fibrosis is lacking. Therefore, exploring the pathogenesis of renal fibrosis and targeted drugs is of great importance. In this review, we provided an overview of current research advances in the mechanisms of renal fibrosis, summarized the promising therapeutic strategies for delaying CKD progression and proposed possible research priorities in the future. First of all, renal fibrosis is characterized by a complex network composed of various cytokines, inflammatory mediators, and signaling pathways. Employing cutting-edge technologies, such as single-cell RNA sequencing for cell-type-specific fibrosis markers and utilizing big data to develop innovative algorithms, could enhance early diagnostic capabilities and guide stratification. In addition, compared with Western medicine, the diverse active ingredients found in traditional Chinese medicine (TCM) have distinct advantages in modulating the networks of renal fibrosis. Therefore, purifying the effective components of TCM and synthesizing the corresponding compounds might become a hotspot in future research. Adaptive design and using human kidney organoid models for drug screening can determine the active ingredients efficiently. Combination regimens may provide better efficacy by reducing the dosage of a single drug and alleviating the side effects. Moreover, epigenetic reprogramming is expected for the complete treatment of renal fibrosis. Finally, how to address the potential toxicity and off-target effects of pathway inhibitors or TCM compounds still remains a challenge.

## GRAPHICAL ABSTRACT

**Figure:**
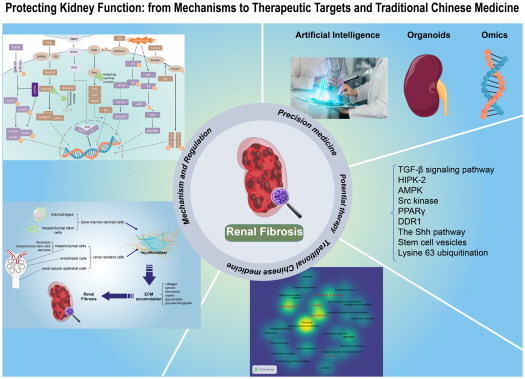

Chronic kidney disease (CKD) is characterized by abnormalities in kidney structure or function that persist for at least 3 months and have significant health implications. It is a major public health concern, affecting approximately 13% of the adult population worldwide, a figure that continues to rise annually. In 2019 alone, over 10 million individuals were newly diagnosed with CKD, and the incidence of CKD-related deaths is also increasing [1]. CKD has become the eighth leading cause of increased disability-adjusted life years (DALYs) among middle-aged and older adults [2]. Furthermore, due to the lack of effective treatments, approximately 11% of patients with stage 3 CKD will eventually progress to end-stage renal disease (ESRD), necessitating kidney transplantation or lifelong dialysis, which not only diminishes patients’ quality of life but also imposes a substantial economic burden on their families and society [3].

Renal fibrosis represents the final outcome of CKD progression to ESRD. The primary pathological alterations include the replacement of normal nephrons with the excessive proliferation of fibroblasts and myofibroblasts, as well as the production and accumulation of extracellular matrix (ECM). These changes lead to tubule atrophy, chronic interstitial inflammation, fibrogenesis, glomerulosclerosis, and vascular rarefaction [4]. Fibrotic tissue is usually regarded as a scar formed after the repair of kidney injury. In cases of mild kidney injury, the deposited ECM can be gradually absorbed and resolved. However, in patients with severe and persistent kidney injury, the accumulated fibrotic substances become resistant to degradation, ultimately disrupting the original kidney structure and causing an irreversible decline in kidney function [3,5]. As research advances, several molecular pathways associated with renal fibrosis have been elucidated, prompting the proposal of drugs targeting these pathways. In this review, we provided an overview of the current research advances in the mechanisms of renal fibrosis, summarized the novel and promising therapeutic strategies for delaying CKD progression and proposed possible research priorities in the future.

## 1. Mechanism insights of renal fibrosis

### 1.1. Sources and roles of myofibroblasts in renal fibrosis

**Figure 1.**
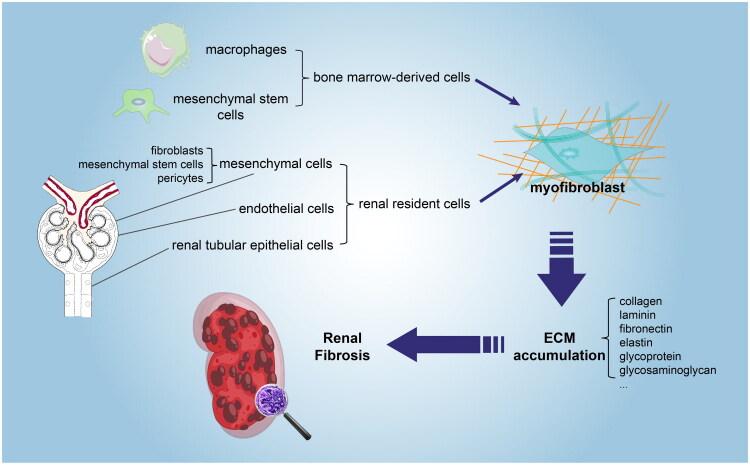
The sources and role of myofibroblast in renal fibrosis.

The ECM, composed of collagen, elastin, laminin, tenascin, proteoglycans, glycosaminoglycans, and hyaluronic acid, is a dynamic three-dimensional macromolecular network. It provides structural support and information transmission for cells and tissues [6]. Present in both the renal cortex and medulla, the ECM is also a major component of the glomerular basement membrane. An ECM deficiency can lead to severe kidney diseases, such as Alport syndrome, in which patients produce aberrant type IV collagen that disrupts the structure and function of the glomerular basement membrane [7]. Conversely, excessive accumulation of ECM in the kidney results in renal fibrosis. Various cell types participate in the synthesis and secretion of the ECM, with myofibroblasts being the most significant contributors [8]. Research suggests that renal fibrosis induced by unilateral ureteral occlusion (UUO) may be alleviated through the specific ablation of proliferating myofibroblasts [9]. Indeed, myofibroblasts are rarely found in healthy kidneys and typically arise from the transformation of other cell types. Pro-fibrotic factors usually stimulate the transformation or differentiation of tubular epithelial cells, endothelial cells, fibroblasts, mesenchymal stem cells, pericytes, and bone marrow-derived cells into myofibroblasts (Figure 1) [10].

The transformation of renal tubular epithelial cells into myofibroblasts represents a type of epithelial-to-mesenchymal transition (EMT). During this process, the cells acquire traits typical of mesenchymal cells, such as alpha-smooth muscle actin (α-SMA) expression and a loose distribution, while losing characteristics associated with epithelial cells, including tight cellular arrangement and E-cadherin expression [11]. EMT is classified into three subtypes based on biological context: type 2 is implicated in organ fibrosis, whereas types 1 and 3 are associated with embryogenesis and cancer invasion, respectively [12]. In the past century, researchers have identified that EMT occurs during the early stage of crescent formation, with myofibroblasts serving as the predominant fibroblasts in the crescent [13]. Although it was previously thought that EMT was the primary source of myofibroblasts in renal fibrosis, more recent studies have indicated that only 5% of myofibroblasts originate from epithelial cells [9,14]. Similarly, endothelial cells in blood vessels can also transform into myofibroblasts, a process known as endothelium-to-mesenchymal transition (EndMT) [15]. Tie2Cre lineage tracing has confirmed the involvement of endothelial cells in renal fibrosis in mouse models, identifying them as one of the sources of myofibroblasts [16]. EndMT is analogous to EMT and can be considered a distinct subtype. However, unlike epithelial cells, endothelial cells downregulate the expression of VE-cadherin, an endothelial cell marker, during this transformation [11].

Myofibroblasts are also derived from renal-resident mesenchymal cells, including fibroblasts, mesenchymal stem cells, and pericytes surrounding arteries [10]. By tracking Gli1, a marker of mesenchymal stem cells, researchers proved that these cells were capable of transforming into myofibroblasts during the fibrosis process in various organs, including the kidney, and that the fibrosis could be alleviated by inhibiting this transformation [17,18]. In recent years, it has become widely accepted that renal-resident mesenchymal stem cells are the primary source of myofibroblasts in renal fibrosis. However, some studies continue to suggest that EMT is the main source, potentially due to variations in research models and detection techniques [19]. Therefore, more rational and precise research on this topic is needed. Because mesenchymal stem cells constitute a relatively small portion of the kidney, some researchers have posited that they do not play a significant role in renal fibrosis. They propose that mesenchymal stem cells might directly induce other cells to differentiate into myofibroblasts through intercellular communication, although there is currently no evidence to support this theory [19].

Researchers have also successfully demonstrated that fibroblasts and pericytes are the sources of myofibroblasts through single-cell RNA sequencing technology [14]. In diabetic nephropathy (DN) mice with renal fibrosis, pericytes were observed to transform into myofibroblasts [20]. Additionally, the *c-Abl* gene and the receptor for activated C-kinase 1 (RACK1) have been found to play significant roles in the differentiation of fibroblasts into myofibroblasts [21]. Furthermore, certain bone marrow-derived cells, such as macrophages and mesenchymal stem cells, also convert into myofibroblasts during renal fibrosis [10,22]. Xu et al. found that mannan-binding lectin (MBL) can inhibit the transformation of macrophages into myofibroblasts during renal fibrosis, with the absence of MBL exacerbating fibrosis [23]. However, myofibroblasts derived from bone marrow differ from those originating from renal-resident cells in that they do not proliferate and contribute minimally to the ECM [9]. Therefore, myofibroblasts derived from bone marrow may have a limited impact on renal fibrosis or may indirectly induce fibrosis through the secretion of specific pro-fibrotic factors [24].

Myofibroblasts and the ECM can be gradually dissolved following minor injuries. However, myofibroblasts persist in cases of prolonged or severe injury, which may be linked to alterations in cellular epigenetics. Bechtel et al. were the first to establish a correlation between epigenetics and renal fibrosis. They discovered that sustained low expression of the Ras protein activator-like 1 (*RASAL1*) gene, following methylation, was associated with the continuous activation of fibroblasts, a finding that was corroborated in patients with CKD [25,26]. Methylation is mediated by the DNA methyltransferases 1. 5′-azacytidine, an inhibitor of DNA methyltransferases 1, can ameliorated renal fibrosis in folic acid induced mice [25]. Another inhibitor, dihydroartemisinin, is believed to reverse renal fibrosis in the adenine-induced CKD mouse model [27]. Similarly, DNA methylation contributes to the persistence of myofibroblasts in renal fibrosis. For example, methylation of the Kruppel-like factor 4 (*KLF4*) gene promotes EMT, while inhibition of disruptor of telomeric silencing 1-like (DOT1L), a histone lysine methyltransferase, impedes EMT [28]. Furthermore, during acute kidney injury (AKI), pericytes exhibit hypermethylation of the *Ybx2* gene, which maintains them in a nonstationary state and renders them susceptible to transformation into myofibroblasts, thereby facilitating the chronicity of AKI [29]. This phenomenon may be linked to the irreversibility of renal fibrosis. In addition to DNA methylation, other factors such as histone modification and non-coding RNA also play significant roles in the development of renal fibrosis [10,30]. Researchers discovered the epigenetic changes induced by hypoxia injury were stored in cells as ‘hypoxic memory’ [31]. Aspartyl-tRNA synthetase anti-sense 1, a lncRNA, was upregulated by binding to the promotor of hypoxia-inducible factor-1 under hypoxic conditons in renal tubular epithelial cells [32]. Similarly, high blood glucose induces persistent epigenetic changes called ‘metabolic memory’, which will not recover even if the blood glucose is well controlled [33].

In conclusion, myofibroblasts serve as the primary source of the substantial ECM that accumulates in renal fibrosis. However, there is ongoing debate about their principal origin. Most research suggests that mesenchymal stem cells residing in the kidney are the predominant source. Additionally, epithelial cells, endothelial cells, pericytes, and bone marrow-derived cells have the capacity to differentiate into myofibroblasts. This transformation is accompanied by epigenetic changes that may perpetuate a state of continuous proliferation and activation in these cells, ultimately leading to the irreversibility of renal fibrosis.

### 1.2. Signaling pathways of renal fibrosis

**Figure 2.**
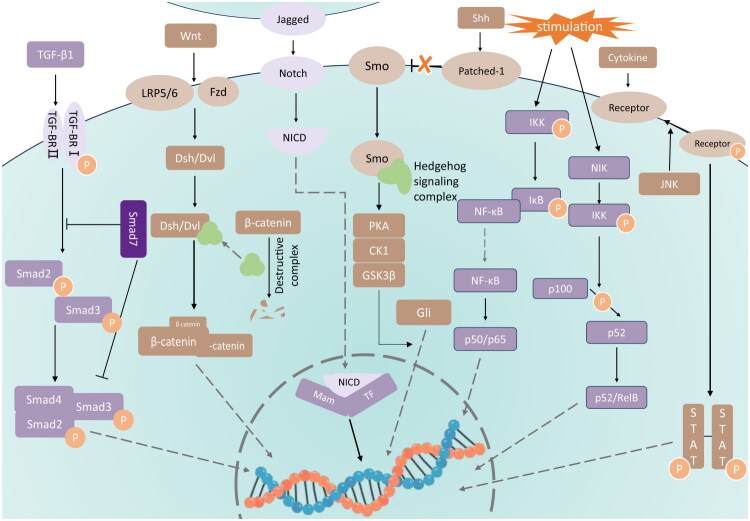
Signaling pathways of renal fibrosis.

The activation of pro-fibrotic signaling pathways leads to the dysregulation of target gene transcription, which is considered one of the primary processes underlying renal fibrosis. Transforming growth factor-β (TGF-β), Wnt, Notch, Hedgehog, Janus kinase (JAK)/signal transducer and activator of transcription (STAT), and nuclear factor kappa B (NF-κB) signaling pathways are widely recognized as being associated with renal fibrosis and thus serve as potential targets for therapeutic intervention (Figure 2). Below, we briefly introduce these six signaling pathways and their roles in the development of renal fibrosis.

#### 1.2.1. The TGF-β signaling pathway

It has been demonstrated that TGF-β promotes CKD and its inhibition alleviates renal fibrosis [34–38]. There are three subtypes of TGF-β, with TGF-β1 being the primary factor in the fibrosis of several organs, including the kidney [39]. In the kidney, TGF-β1 regulates the expression of target genes through both classical and non-classical signaling pathways. This regulation promotes the proliferation and differentiation of fibroblasts and the production of ECM, as well as EMT and EndMT, ultimately leading to renal fibrosis [35].

In the classical pathway, TGF-β1 binds to transforming growth factor-β receptor II (TGF-βRII), which activates transforming growth factor-β receptor I (TGF-βRI, also known as ALK5). Once activated, TGF-βRI phosphorylates Smad2 and Smad3, leading to the formation of a trimer with Smad4. This trimer subsequently translocates into the nucleus and binds to specific promoters to regulate the expression of target genes [35,40]. Additionally, Smad7, an inhibitory Smad, interferes with the signaling pathway by competing with the Smad2/3/4 trimer for binding sites on target genes [41,42]. As research has developed, the downstream RNAs of this signaling pathway have been progressively identified. The TGF-β/Smad pathway upregulates the expression of microRNA-21 (miR-21), which promotes renal fibrosis by modulating various metabolic pathways. For example, miR-21 directly inhibits peroxisome proliferator-activated receptor-α (PPARα) and MPV17-like proteins; the former prevents renal fibrosis by regulating lipid peroxidation, while the latter reduces the generation of reactive oxygen species [43]. Additionally, miR-21 inhibits Smad7, further enhancing the pathway and triggering autophagy [44]. Furthermore, the TGF-β/Smad pathway also upregulates other pro-fibrotic microRNAs, such as miR-17-5p, miR-150-5p, miR-216a, miR-217, miR-382, miR-433, and miR-495-5p, while downregulating miR-19b, miR-26a, miR-29, miR-30, miR-101, miR-130b, miR-374a-5p, and other inhibitory microRNAs [35,45–47]. Some microRNAs with bidirectional effects are also targeted by the TGF-β/Smad pathway, including miR-145, miR-192, miR-200, and Let-7 [35,48]. Additionally, the TGF-β/Smad pathway promotes renal fibrosis by targeting specific long-chain non-coding RNAs (lncRNAs), including lncRNA np_5318/np_17856 [49].

Bone morphogenetic protein (BMP) belongs to the TGF-β family, and BMP-7 can block the TGF-β signaling pathway, inhibit the accumulation of ECM, and play an anti-fibrotic role. Upon binding to its receptor, BMP-7 reduces the formation of the Smad2/3/4 trimer by activating downstream Smad1/5/8, which subsequently binds to Smad4. Furthermore, BMP-7 enhances the expression of Ski-related novel protein N (SnoN), a negative regulator of the TGF-β/Smad pathway through Smad1/5 [50]. Additionally, BMP-7 inhibits the PI3K/Akt pathway, which is considered a non-classical TGF-β pathway [51]. However, it is important to note that BMP-7 does not alleviate aristolochic acid-induced renal fibrosis [52]. Therefore, the inhibitory effect of BMP-7 on renal fibrosis may be somewhat limited.

In addition to the classical pathway, TGF-β can also transmit fibrotic signals through non-classical pathways, including phosphatidylinositol 3-kinase (PI3K)/protein kinase B/mammalian target of rapamycin (mTOR), p38/c-Jun N-terminal kinase (JNK), extracellular regulated protein kinase (ERK)/mitogen-activated protein kinase (MAPK), and Ras homologous gene family member A (RhoA). These pathways not only directly mediate transcription factors but also indirectly regulate target genes by modulating the Smad complex [35,53]. The activation of the p38/JNK pathway relies on the autoubiquitination of TNF receptor-associated factor 6 (TRAF6) at lysine 63, a process facilitated by the TGF-β receptor [54]. However, Smad6, another inhibitory Smad, influences TRAF6 autoubiquitination by recruiting deubiquitinating enzymes, thereby inhibiting the p38/JNK pathway [54].

#### 1.2.2. The Wnt signaling pathway

Wnt proteins are a class of secreted glycoproteins that influence numerous biological processes in the human body, including embryonic development, tissue homeostasis, and disease onset [55]. The Wnt signaling pathway also plays a critical role in regulating renal fibrosis [55,56]. Extracellular Wnt protein binds to Frizzled (Fzd) family receptor on the target cell membrane and activates Disheveled (Dsh/Dvl) protein, along with the co-receptor low-density lipoprotein receptor-related protein 5/6 (LRP5/6) [57]. Following this activation, there is a significant accumulation of β-catenin within the cell. This occurs because the scaffold protein axin, which is a component of the ‘destructive complex’ of β-catenin, is recruited by the activated Dsh/Dvl protein in the classical pathway [58,59]. Subsequently, β-catenin enters the nucleus and binds to T cell factor-lymphoid enhancer factor (TCF-LEF), enhancing the expression of pro-fibrotic genes [60]. In addition to this classical pathway, Wnt can also regulate the expression of fibrosis-related target genes through several non-classical pathways, including Wnt/PCP and Wnt/Ca^2+^ signaling pathways [61].

The Wnt/β-catenin signaling pathway is activated in a renal fibrosis model induced by UUO [62]. In this context, secreted frizzled-related protein 4 (sFRP4), a negative regulator of the Wnt/β-catenin signaling pathway, reduces levels of fibronectin, α-SMA, and myofibroblasts in the kidney, thereby alleviating renal fibrosis in mouse models. Furthermore, activation of the Wnt signaling pathway has also been observed in other renal fibrosis models, including DN, aging, and herbicide atrazine-induced models [63–65]. At the cellular level, Wnt signaling from tubular epithelial cells influences fibroblasts, promoting their proliferation and differentiation into myofibroblasts [65,66].

Additionally, the Wnt signaling pathway induces the production of TGF-β in aging tubular epithelial cells and promotes the TGF-β pathway [65]. At the genetic level, numerous target genes of the Wnt signaling pathway have been strongly associated with fibrosis, like fibronectin, MMP-7, plasminogen activator inhibitor-1(PAI-1), SNAIL, renin-angiotensin system (RAS) and hepatocyte growth factor (HGF) [57]. Fibronectin is the main component of ECM. MMP-7 is a protease that can degrade ECM. However, it can aggravate renal fibrosis by increasing the expression of β-catenin, forming a vicious cycle [67]. PAI-1 is an inhibitor of fibrinolysis and can promote ECM accumulation [68]. The SNAIL gene is a transcription factor of E-cadherin which is involved in EMT [69]. RAS has been proven to be associated with age-related mitochondrial dysfunction and renal fibrotic lesions because all RAS genes have the binding sites of TCF-LEF [70]. HGF exerts anti-fibrotic effects by inhibiting TGF-β and renal inflammation [57]. The Wnt signaling pathway can down-regulate its expression. Moreover, this pathway is bidirectional. It plays a repair role in AKI, however, its persistent activation leads to CKD and renal fibrosis [71].

#### 1.2.3. The Notch signaling pathway

The Notch signaling pathway plays a crucial role in cell differentiation, apoptosis, proliferation, and other cellular processes. Upon binding to a ligand located on the membrane of an adjacent cell, the transmembrane receptor Notch undergoes a cleavage reaction that releases the Notch intracellular domain (NICD). The NICD then translocates to the nucleus and forms a trimer with transcription factors and co-activators, such as Mastermind (Mam), which can activate the expression of profibrotic genes [72]. Under physiological conditions, the Notch signaling pathway is essential for kidney development because any segment of nephrogenesis would be inhibited when the pathway is disrupted, while there is little Notch expression in the kidneys of healthy adults [73,74]. Currently, it has been proven that the Notch signaling pathway is activated in renal fibrosis. Notch expression is elevated in a renal fibrosis model induced by UUO, and mice lacking Notch3 exhibited milder kidney damage compared to wild-type mice [75]. Furthermore, the Notch signaling pathway is involved in renal fibrosis induced by folic acid, DN, and polycystic kidney disease [76–79]. Notch2 and its ligand Jagged1 have been demonstrated to exhibit a strong correlation with renal fibrosis in genomic analyses of human kidney samples [79]. A study indicated that the Notch pathway induced oxidative damage and mitochondrial dysfunction to aggravate renal fibrosis [80]. SNAIL genes are also the target of the Notch pathway [81]. In addition, Notch and Jagged1 are mainly expressed on podocytes and their expression levels are correlated with the advance of renal fibrosis [82]. Therefore, the Notch pathway mainly influences the function of podocytes. The Notch signaling pathway was regulated by TGF-β, as the expression of Notch1 and Notch3 in mesangial cells increased after exposure to TGF-β1 for 30 min [75].

#### 1.2.4. The Shh signaling pathway

Mammals possess three types of Hedgehog ligands: Sonic hedgehog (Shh), Indian hedgehog (Ihh), and Desert hedgehog (Dhh). Shh is extensively expressed in humans and linked to kidney development and repair [83]. In the classical signaling pathway, Shh binds to the membrane receptor Patched-1, which inhibits the pathway activator Smo protein, through autocrine or paracrine [83]. Moreover, the Smo activation depends on the localization of cilia. Therefore, the absence of cilia on the cell membrane can lead to the inhibition of the Shh pathway [58].

Smo protein binds to the Hedgehog signaling complex and facilitates the translocation of the transcription factor Gli (primarily Gli2) into nucleus *via* three kinases: PKA, CK1, and GSK3β. This process ultimately regulates the expression of target genes [17,83,84]. Research has demonstrated that the Shh signaling pathway primarily targets fibroblasts to enhance their proliferation and ECM deposition during renal fibrosis [85]. The downstream of the pathway involves SNAIL, α-SMA, fibronectin, collagen I, and desmin which are all profibrotic factors [86]. Hh ligands are mainly produced by renal tubular epithelial cells, and the responding cells are interstitial fibroblasts and pericytes [87].

#### 1.2.5. The JAK/STAT signaling pathway

JAKs are a family of non-receptor tyrosine kinases that include four subtypes [88]. The JAK/STAT signaling pathway is initiated by the cytokines binding to their respective receptors. JAK phosphorylates receptors, which subsequently bind to STATs, leading to their phosphorylation and dimerization. These dimers then translocate to the nucleus and regulate the transcription of target genes [89]. The JAK/STAT signaling pathway is activated in both UUO and ischemia-reperfusion injury (IRI) induced renal fibrosis models [90,91]. Moreover, a study suggests that this pathway may play a crucial role primarily in the early stage of renal fibrosis. Early inhibition of the JAK/STAT pathway can improve renal injury, however, inhibition of this pathway 3 h after injury has no beneficial effects [91]. Additionally, the JAK/STAT signaling pathway is implicated in renal fibrosis associated with DN and glomerulonephritis [89].

#### 1.2.6. The NF-κB signaling pathway

NF-κB is a member of the Rel family of transcription factors, including p50/p105 (NF-κB1), p52/p100 (NF-κB2), RelB, c-Rel, and p65 (RelA) [92]. The precursors of p50 and p52 are p105 and p100, respectively [92]. It is widely accepted that the NF-κB signaling pathway promotes proliferation and inflammation as well as inhibiting apoptosis [93]. The NF-κB pathway comprises both canonical and non-canonical pathways. The canonical pathway is initiated by the phosphorylation of inhibitor of kappa B kinase (IKK). IKK subsequently degrades inhibitor of kappa B (IκB), which normally binds to NF-κB and prevents its translocation to the nucleus. This allows p50/p65 to translocate to the nucleus and regulate the expression of target genes [93]. In contrast, the activation of NF-κB-inducing kinase (NIK) initiates the non-canonical pathway. NIK phosphorylates IKK, which subsequently leads to the conversion of p100 into p52. The resulting p52 then forms heterodimers with RelB, and the p52/RelB complex enters the nucleus and regulates expression of target genes which are primarily cytokines [93]. The NF-κB signaling pathway is activated in renal fibrosis induced by various mechanisms, including UUO, IgA nephropathy, membranous nephropathy, DN, hypertensive nephropathy, and kidney transplantation [93–98].

Therefore, the transmission of pro-fibrotic signals in kidneys does not rely on a single pathway. On the contrary, it involves multiple pathways that affect the expression of various target genes. There are differences among various signal pathways, and each pathway has its own unique attributes. For example, the Wnt/β-catenin pathway is bidirectional; the Notch pathway mainly acts on podocytes; the Shh pathway mainly acts on interstitial cells and pericytes; the JAK/STAT pathway is activated when injury occurs; the NF-κB pathway mainly mediates inflammatory responses. Collectively, these elements form a complex network that ultimately contributes to the development of renal fibrosis.

## 2. Therapeutic interventions for renal fibrosis

### 2.1. Potential therapeutic targets and drugs for renal fibrosis

#### 2.1.1. TGF-β signaling pathway

A molecule targeting both the fibronectin extra domain A isoform and TGF-β was constructed with dual variable domain Ig (DVD-Ig) technology, successfully reducing collagen deposition in the UUO mouse model and alleviating renal fibrosis [99]. Fresolimumab, a monoclonal antibody against TGF-β, inhibits TGF-β signal transmission, leading to an anti-fibrotic effect. In a clinical trial of fresolimumab for hormone-insensitive primary focal segmental glomerulosclerosis (NCT01665391), the drug demonstrated good safety and tolerability. However, compared with the placebo, it did not significantly reduce proteinuria [100,101]. In addition, researchers found that Fresolimumab could stabilize eGFR, indicating its effect of improving renal fibrosis was independent of proteinuria reduction [101]. TGF-β1-specific monoclonal antibodies LY2382770 failed to improve renal function in a clinical trial on DN (GC1008FSGS00505) [102]. Due to the multiple physiological functions of TGF-β, its direct inhibition may lead to various adverse reactions, which limit the dosage and usage duration of such drugs and results in unsatisfactory clinical trial results [103]. Pirfenidone, a medication for pulmonary fibrosis, has been shown to suppress TGF-β production and delay the decline of eGFR in clinical trials involving patients with focal segmental glomerulosclerosis [104]. Cell division autoantigen 1 (CDA1) is an anti-proliferative protein which can regulate the TGF-β/Smad3 signaling pathway [105]. Knockout of *CDA1* genes was found to reduce TGF-β/Smad3 signal transduction, indicating CDA1 targeting could improve DN-induced renal fibrosis in a relatively safe manner [105]. The ECM accumulation decreased by 60% in diabetic mice treated with a CDA1 inhibitor CHA-061 than that with vehicle treatment [105]. Therefore, CDA1 may represent a promising therapeutic target for renal fibrosis.

Angiotensin II upregulates TGF-β1 expression in various cell types, suggesting that angiotensin II receptor blockers (ARBs) may have anti-fibrotic effects. A case report demonstrated that the topical application of losartan alleviates corneal cicatricial fibrosis [106]. Additionally, losartan improves renal fibrosis after transplantation by inhibiting the expression of miR-21 and TGF-β in kidney transplant patients [107]. Similarly, imidapril, an angiotensin-converting enzyme inhibitor (ACEI), inhibits TGF-β signaling and mitigates renal fibrosis in DN mice [108]. Furthermore, silymarin, a drug used to enhance liver function, exhibits an anti-tubulointerstitial fibrotic effect, potentially through inhibition of the TGF-β/Smad signaling pathway [109].

THR-123 is an oral formulation that inhibits the TGF-β signaling pathway by activating the BMP7 receptor Alk3, which is predominantly expressed in renal tubular epithelial cells. This compound has anti-fibrotic effects on UUO, Alport syndrome, and DN mouse models [110]. Further studies indicated that the combination of THR-123 with captopril significantly inhibits renal tubular atrophy and dilation and protects renal function in DN mice [110]. THR-184, a BMP7 analog, exhibited limited efficacy in a clinical trial aimed at preventing AKI associated with cardiopulmonary bypass during cardiac surgery. However, it may offer a novel approach for the prevention of renal fibrosis [111].

The non-classical TGF-β signaling pathway represents a potential target for the treatment of renal fibrosis. mTOR serves as a crucial link in this non-classical pathway, and its inhibitors (everolimus, rapamycin, and sirolimus) are currently employed clinically as immunosuppressants. Notably, among kidney transplant recipients, the IF/TA score of the transplanted kidneys in the everolimus group was lower [112]. In patients with idiopathic pulmonary fibrosis, sirolimus has been observed to decrease both the blood levels of fibrocytes and the expression of α-SMA [113]. Furthermore, mTOR inhibitors have demonstrated anti-fibrotic effects in various animal models of renal fibrosis, including the UUO, 5/6 nephrectomy, and tacrolimus induction models [114–116]. Consequently, mTOR inhibitors may represent a promising new approach for the treatment of renal fibrosis.

Renal fibrosis may be mitigated by inhibiting integrins, which act as activators of TGF-β. MK-0429, a broad integrin inhibitor, has been shown to improve fibrosis in the DN mouse model by reducing the expression of fibrosis-related genes induced by TGF-β, as well as by decreasing renal collagen accumulation [117].

The TGF-β signaling pathway features a positive feedback mechanism. Initially, TGF-β induces a rapid increase in NUAK family kinase 1 (NUAK1). Subsequently, NUAK1 enhances TGF-β/Smad signaling and the activity of the transcription cofactor Yes-associated protein (YAP). In turn, YAP, together with the transcriptional coactivator TAZ (transcriptional coactivator with PDZ-binding motif), promotes the expression of the *NUAK1* gene [118]. Consequently, inhibition of NUAK1 can significantly disrupt this pathway. The NUAK1 inhibitor HTH-02-006 has been demonstrated to reduce renal fibrosis markers, such as type I collogen and α-SMA, in the UUO mouse model [118].

#### 2.1.2. Homeodomain-interacting protein kinase 2

Homeodomain-interacting protein kinase 2 (HIPK-2) is a serine/threonine kinase involved in various pro-fibrotic signaling pathways, including TGF-β/Smad, Wnt/β-catenin, and Notch [119]. BT173, a synthetic compound, specifically targets the interaction between HIPK-2 and Smad3, thereby inhibiting the TGF-β/Smad signaling pathway without affecting the original kinase activity of HIPK-2. This compound has been shown to reduce fibrosis in both UUO and AIDS-related nephropathy mouse models, and improve renal function and reduce proteinuria [120]. Additionally, niclosamide, an oral agent used to treat intestinal worms, also inhibits HIPK-2 and alleviates renal fibrosis in mice induced by adriamycin and UUO *via* modulating multiple pro-fibrotic pathways [121].

#### 2.1.3. Adenosine monophosphate-activated protein kinase

Adenosine monophosphate-activated protein kinase (AMPK) plays a crucial role in regulating cellular energy metabolism and mitigating oxidative stress. Research has demonstrated that the activation of AMPK can upregulate mitophagy and renal tubular epithelial autophagy, thereby reversing renal fibrosis [122]. 5-Aminoimidazole-4-carboxamide-1-β-D-ribofuranoside (AICAR), an AMP analog and AMPK agonist, has been shown to reduce renal interstitial fibrosis and ECM accumulation by inhibiting TGF-β-mediated myofibroblast activation in UUO mice [123]. However, its application is limited due to the low bioavailability. Although AICAR analogs have been developed, further studies are necessary to elucidate their anti-fibrotic effects and bioavailability [124]. Metformin, widely used for diabetes, also acts as an AMPK activator. In the CKD model induced by subtotal nephrectomy, metformin has been found to reduce more than half of proteinuria and markers of renal fibrosis compared to untreated rats, independent of blood glucose and blood pressure levels [125].

Adiponectin can induce AMPK activation. AdipoRon, an oral adiponectin receptor agonist, has been shown to inhibit EMT and improve renal fibrosis in the hypertensive mouse model [126]. Furthermore, liver kinase B1 (LKB1), a kinase that activates AMPK, may serve as a potential target for renal fibrosis therapy. Research indicates that the piericidin analog S14, an LKB1 agonist, can delay mouse renal fibrosis *via* mediating autophagy and maintaining mitochondrial homeostasis [127].

#### 2.1.4. Src kinase

Src kinase is an intracellular tyrosine kinase involved in multiple processes associated with renal fibrosis, including the promotion of macrophage–myofibroblast transformation and fibroblast proliferation and the inhibition of extracellular matrix-degrading enzyme MMP-2 [122]. KF-1607, a low-toxicity Src kinase inhibitor, has been synthesized and demonstrated to alleviate renal interstitial fibrosis in a mouse model [128].

#### 2.1.5. Peroxisome proliferator-activated receptor γ

Peroxisome proliferator-activated receptor γ (PPARγ) has been identified as having a renal protective effect, which may be due to its regulation of renal energy metabolism and TGF-β signaling [129]. Thiazolidinediones, such as rosiglitazone and pioglitazone, are a class of PPARγ agonists that can be used in the treatment of type 2 diabetes as insulin sensitizers. In animal studies, rosiglitazone inhibits interstitial fibrosis induced by uric acid nephropathy, and its bioavailability can be enhanced using drug delivery systems, such as nanotechnology, resulting in improved anti-renal fibrotic effects [130,131]. Saroglitazar, which is used to treat nonalcoholic steatohepatitis, is a PPARα/γ agonist that has been shown to inhibit inflammatory responses and interstitial fibrosis in the UUO mouse model [132]. A novel drug, RB394, which both activates PPARγ and inhibits soluble epoxide hydrolase, demonstrated a more significant anti-fibrotic effect in a mouse model than rosiglitazone alone, likely because soluble epoxide hydrolase has been shown to promote renal fibrosis [133].

#### 2.1.6. Discoidin domain receptor 1

Discoidin domain receptor 1 (DDR1) is a tyrosine kinase receptor that is activated by collagen. It promotes fibrosis by regulating cell–collagen interactions, increasing β-catenin activity, and stimulating TGF-β secretion [122]. Compound 2.45, a specific DDR1 inhibitor identified in the DNA-Encoded Compound Library, demonstrates favorable inhibitory effects both *in vivo* and *in vitro* and successfully ameliorates renal fibrosis in a preclinical mouse model of Alport syndrome [134].

#### 2.1.7. The Shh pathway

The transcription factor Gli2 plays a major role in regulating the Shh pathway. Darinaparsin, a drug currently in clinical trials for leukemia, has been shown to antagonize Gli2 and halt the cell cycle of myofibroblasts, thereby alleviating renal fibrosis in a mouse model [18]. Similarly, another Gli antagonist, GANT61, has been demonstrated to improve renal fibrosis in UUO and polycystic kidney mice [135].

#### 2.1.8. Stem cell vesicles

Mesenchymal stem cell vesicles have been shown to alleviate fibrosis in various models, such as DN and UUO mice, probably due to their intravesicular content of anti-fibrotic biological molecules, including RNA, proteins, and lipids [136–139]. In a renal fibrosis mouse model induced by aristolochic acid, human liver stem cell vesicles may reduce collagen fibers in the tubulointerstitial region by regulating β-catenin signaling [140]. The clinical trials on Mesenchymal Stromal Cell Therapy in Renal Recipients have entered Stage II [141]. Furthermore, mesenchymal stem cell vesicles have been used as drug delivery carriers. For example, mesenchymal stem cell vesicles modified with superparamagnetic iron oxide nanoparticles (SPION) can accumulate in the kidney when exposed to specific magnetic fields, thereby enhancing their anti-fibrotic effects [142]. Vesicle treatment can reduce the renal pathological damage score from 70% to 26%.

#### 2.1.9. Lysine 63 ubiquitination

The lysine 63 ubiquitination process has been associated with DN fibrosis. Under high-glucose conditions, renal tubular epithelial cells overexpress ubiquitin-conjugating E2 enzyme variant 1 (UBE2v1), which increases the levels of lysine 63-ubiquitinated proteins in the cells and elevates intracellular α-SMA. This alteration affects the distribution of actin, a key component of the cellular scaffold, resulting in tubular atrophy and renal interstitial fibrosis [143]. The UBE2v1 inhibitor NSC697923 has been shown to reduce α-SMA and collagen expression in DN mice, and the combination of NSC697923 with an ACEI produces a more pronounced anti-fibrotic effect in animal models [143,144].

### 2.2. Traditional chinese medicine (TCM) and its active ingredients

**Figure 3.**
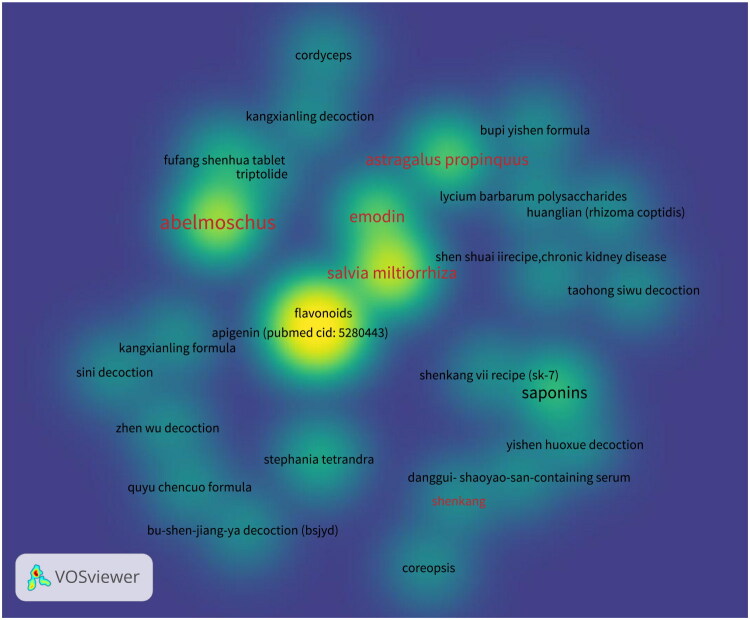
The density visualization of the keywords which are the names of TCM.

There is a substantial body of research on the treatment of renal fibrosis using traditional Chinese medicine (TCM) and its active ingredients. An analysis of keywords related to renal fibrosis and TCM published in the past 5 years, conducted using VOSviewer software on data retrieved from the PubMed database, revealed that *Astragalus propinquus*, *Abelmoschus manihot*, *Salvia miltiorrhiza*, rhubarb, and Shenkang injection have been the primary research focuses (Figure 3). The following section will introduce the research progress concerning these five TCMs.

*Astragalus propinquus* has been shown to improve fibrosis in various organs, with several active ingredients contributing to this effect [145]. Huangqi Decoction showed to improve renal fibrosis through inhibiting the Wnt/β-catenin pathway in UUO mice [146]. Research indicates that astragalus polysaccharide may alleviate renal fibrosis in DN mice by targeting the lncRNA Gm41268/PRLR pathway to promote autophagy [147]. Mullein isoflavone, a component of *Astragalus*, enhances the inhibitory effect of mesenchymal stem cells on podocyte apoptosis, thereby helping to protect the kidneys in mouse tests [148]. Astragaloside IV regulates the MAPK and NF-κB signaling pathways and inhibits fibroblast differentiation and ECM formation in the renal fibroblasts treated with TGF-β1 [149]. It has also been proven to inhibit pyroptosis of renal tubular epithelial cells and alleviate UUO rats renal fibrosis by regulating the cAMP/PKA signaling pathway [150]. In addition, Huangqi Chifeng Decoction, a traditional Chinese herbal remedy containing *Astragalus*, has been shown to reduce urinary protein and mitigate renal fibrosis by inhibiting TGF-β in patients with immunoglobulin A nephropathy [151].

Huangkui capsule is widely used in clinical settings and has been shown to effectively reduce urinary protein levels. The primary ingredient in Huangkui capsule is *Abelmoschus manihot*. This formulation has the potential to ameliorate renal fibrosis, with five active components of *Abelmoschus manihot*—quercetin, hypericin, isoquercetin, lignin, and quercetin-3′-O-glucoside—demonstrated to inhibit hyperglycemia-induced EMT and renal interstitial fibrosis in DN mice [152]. Quercetin has shown anti-fibrotic effects through various targets in many organs. In UUO mice, quercetin inhibits fibroblast activation and the expressions of α-SMA and fibronectin through a combined inhibition of mTOR and β-catenin signaling pathways [153]. Quercetin can also bind to Alk5 and impairing its activity [154]. The effect of reducing epidural fibrosis by quercetin is supposed to be related to the PI3K/Akt/mTOR signaling pathway in rats [155]. Additionally, the total flavones derived from *Abelmoschus manihot* have been found to enhance renal function and mitigate fibrosis in DN rats by targeting TGF-β, necrotic apoptosis, and other pathways [156].

Salvianolic acid B, the active ingredient in *Salvia miltiorrhiza*, has been shown to alleviate kidney fibrosis. Its oral absorption and distribution within the kidney can be enhanced by utilizing nanoparticles, thereby improving its anti-fibrotic efficacy [157]. Similarly, salvianolic acid A inhibits renal fibrosis by suppressing the TGF-β1/Smad signaling pathway in rats [158]. Tanshinone, another active ingredient in *Salvia miltiorrhiza*, may improve DN fibrosis in animal models by regulating various signaling pathways, including TGF-β, Wnt/β-catenin, and Notch [159]. In addition, it has been proven that Tanshinone IIA attenuates renal fibrosis in AKI mice through downregulting TGF-β induced fibrocytes recruitment [160]. Guanxinning, a Chinese patent medicine derived from *Salvia miltiorrhiza* for treating angina pectoris, also alleviates renal fibrosis in mice with heart failure [161].

Rhein is one of the main active components of rhubarb. Previous studies have suggested that it may alleviate renal fibrosis in UUO mice by regulating the Shh-Gli1-SNAIL and TGF-β signaling pathways [162,163]. It can ameliorate autophagy and attenuate renal fibrosis in rats with adenine-induced renal tubular injuries by regulating the AMPK/mTOR Signaling [164]. A recent study indicated that rhein also has epigenetic effects, such as reducing the methylation of the Klotho protein promoter and increasing the expression of the histone deacetylases SIRT1 and SIRT3, thereby mitigating kidney fibrosis [165–167]. Emodin, another active component of rhubarb, can alleviate renal fibrosis in animals models, and the mechanism may be related to the p38/MAPK, TGF-β/Smad, and BMP7 pathways [168–170]. Finally, chrysophanol ameliorates renal fibrosis in the UUO mouse model by down-regulating the TGF-β/Smad signaling pathway and directly inhibiting the NKD2/NF-κB pathway [171,172].

Shenkang injection, which primarily comprises rhubarb, *Salvia miltiorrhiza*, *Safflower*, and *Astragalus propinquus*, has been used in the treatment of CKD. It can alleviate kidney fibrosis in UUO mice and is dose-dependent [173]. The efficacy has also been demonstrated in a phase IV clinical observation study [174]. Recently, a study successfully established pharmacokinetic and pharmacodynamic models for several active ingredients in Shenkang injection, including hydroxy saffron yellow pigment A, tanshinol, rheum emodin, and astragaloside IV. This research confirmed that these components exhibit stable drug metabolism characteristics and can improve renal fibrosis [175]. These findings may have significant implications for the future application of the active ingredients in Shenkang injection to the treatment of renal fibrosis.

In recent years, the treatment of renal fibrosis has garnered significant attention. Though various pharmacological agents have demonstrated efficacy in improving renal fibrosis in animal studies, few drugs are approved, which makes it extremely challenging to achieve satisfactory results in clinical trials (Table 1). This discrepancy may be due to short medication duration, small sample size, low drug bioavailability, and restricted administration for safety reasons. Therefore, the design of clinical trials needs to be adjusted. Furthermore, the problem of low bioavailability needs to be addressed through means such as chemical modification of drugs, targeted drug delivery, and combined therapy.

**Table 1.** Drugs used for treating kidney fibrosis.

Drug	Targets	Experimental subjects	Efficacy	References
Clinical trials				
Fresolimumab	TGF-β	Hormone-insensitive primary focal segmental glomerulosclerosis patients	Good safety and tolerability but a less significant reduction in urinary protein	[100,101]
Ly2382770	TGF-β1	DN patients	No significantly improving renal function	[102]
Pirfenidone	TGF-β	Focal segmental glomerulosclerosis patients	Delaying the eGFR decrease	[104]
Losartan	angiotensin II receptor	Kidney transplant patients	Decreasing the tissue expression of miR-21 and TGF-β	[107]
Thr-184	BMP7	Patients after cardiopulmonary bypass pumps during cardiac surgery	No statistically significant effects	[111]
Everolimus	mTOR	Renal transplantation recipients	Decreasing IF/TA score	[112]
Mesenchymal stromal cell therapy	NA^a^	Renal transplantation recipients	NA	[142]
Animal experiments				
TGF-β + fibronectin DVD-Ig	fibronectin extra domain A isoform and TGF-β	UUO mice	Highly location to the fibrotic kidney and decreasing collagen deposition	[99]
Cha-061	CDA1	DN mice	Decreasing ECM accumulation	[105]
Imidapril	angiotensin-converting enzyme	DN mice	Inhibiting TGF-β signaling and decreasing the level of α-SMA, collagen I and fibronectin	[108]
Silymarin	NA	DN mice	Decreasing 24h-uninary proteins and serum creatine, improve the renal fibrosis pathology, and regulating TGF-β/Smad signaling pathway	[109]
Thr-123	Alk3	UUO, Alport syndrome, and DN mice	Anti-fibrotic effects	[110]
Mk-0429	integrins	DN mice	Reducing the expression of fibrosis-related genes induced by TGF-β, and decreasing renal collagen accumulation	[117]
Hth-02-006	NUAK1	UUO mice	Reducing renal fibrosis markers, like type I collogen and α-SMA	[118]
Bt173	HIPK-2 and Smad3	UUO and AIDS-related nephropathy mouse models	Improving renal pathology, and reducing urinary protein	[120]
Niclosamide	HIPK-2	Mouse renal fibrosis mice induced by adriamycin and UUO	Reducing proteinuria, glomerulosclrotic lesions, and interstitial fibrosis	[121]
Aicar	AMPK	UUO mice	Reduce renal interstitial fibrosis and ECM accumulation	[123]
Metformin	AMPK	Subtotal nephrectomy mice	Reducing markers of renal fibrosis and more than half of proteinuria	[125]
Adiporon	adiponectin	Hypertensive mice	Inhibiting EMT	[126]
S14	LKB1	Adriamycin nephropathy mice, 5/6 nephrectomy mice, and UUO mice	Delay renal fibrosis by mediating autophagy and maintaining mitochondrial homeostasis	[127]
Kf-1607	Src kinase	UUO mice	Decreasing α-SMA, collagen I and IV protein expression	[128]
Rosiglitazone	PPARγ	High uric acid nephropathy mice	Inhibiting TGF-β and NF-κB pathway activation, preserving renal function, decreasing urine microalbumin	[130]
Saroglitazar	PPARα/γ	UUO mice	Inhibiting inflammatory responses and interstitial fibrosis	[132]
Rb394	PPARγ and sHE	Mouse	Stronger anti-fibrosis effect than Single-target therapy	[133]
Compound 2.45	DDR1	Preclinical mouse models of Alport syndrome	Preserving renal function and reducing tissue damage	[134]
Darinaparsin	Gli2	UUO mice	Halting the cell cycle of myofibroblasts	[18]
Gant61	Gli	UUO and PKD mice	Inhibiting cyst progression and fibrosis and preserving kidney function	[135]
Human liver stem cell vesicles	NA	Aristolochic acid-induced mice	Reducing the presence of collagen fibers in the interstitial space between tubules	[140]
Mesenchymal stem cell vesicles modified with SPION	NA	UUO rats	Decreasing damage score of renal pathology from 70% to 26%,	[142]
Nsc697923	UBE2v1	DN mice	Reducing α-SMA and collagen expression	[143]

^a^ NA: Not Applicable.

Concurrently, the multi-component and multi-target characteristics of TCM offer promising avenues for treating renal fibrosis, with the identification of active ingredients potentially enhancing therapeutic outcomes while minimizing toxicity and adverse effects (Table 2). However, there are still some issues in the treatment of renal fibrosis with the active ingredients of TCM, such as the lack of long-term and large-scale clinical trials to verify its efficacy; incompletely clear mechanism; difficulty in standardization for mass production.

**Table 2.** The TCM and its active ingredients with anti-fibrotic effects.

TCM	Active ingredients	Targets	Experimental subjects	References
Huangqi Decoction	NA^a^	Wnt/β-catenin pathway	UUO mice	[146]
*Astragalus propinquus*	NA	lncRNA Gm41268/PRLR	DN mice	[147]
*Astragalus propinquus*	Mullein isoflavone	mesenchymal stem cells	Adriamycin induced focal segmental glomerulosclerosis mice	[148]
*Astragalus propinquus*	Astragaloside IV	MAPK and NF-κB signaling pathways	Mouse renal fibroblasts treated with TGF-β1	[149]
*Astragalus propinquus*	Astragaloside IV	cAMP/PKA signaling pathway	UUO rats	[150]
Huangqi Chifeng Decoction	NA	TGF-β	Immunoglobulin A nephropathy patients	[151]
*Abelmoschus manihot*	Quercetin, Hypericin, Isoquercetin, Lignin, and Quercetin-3′-O-glucoside	EMT	DN mice	[152]
*Abelmoschus manihot*	Quercetin	mTOR and β-catenin signaling pathway	UUO mice	[153]
*Abelmoschus manihot*	Quercetin	Alk5	Skin fibrosis animal models	[154]
*Abelmoschus manihot*	Quercetin	PI3K/Akt/mTOR signaling pathway	Epidural fibrosis rats	[155]
*Abelmoschus manihot*	Total flavones	TGF-β, necrotic apoptosis,	DN mice	[156]
*Salvia miltiorrhiza*	Salvianolic acid A	TGF-β1/Smad signaling pathway	5/6 nephrectomy rats	[158]
*Salvia miltiorrhiza*	Tanshinone	TGF-β, Wnt/β-catenin, and Notch	DN models	[159]
*Salvia miltiorrhiza*	Tanshinone IIA	TGF-β induced fibrocytes recruitment	Mice with renal fibrosis after AKI	[160]
Guanxinning	NA	SLC7A11/GPX4 axis	Mice with heart failure	[161]
Rhubarb	Rhein	Shh-Gli1-SNAIL and TGF-β signaling pathways	UUO mice	[162,163]
Rhubarb	Rhein	AMPK/mTOR Signaling	Rats with adenine-induced renal tubular injury	[164]
Rhubarb	Rhein	SIRT1	UUO mice	[165]
Rhubarb	Rhein	SIRT3	5/6 nephrectomy rats	[166]
Rhubarb	Rhein	Klotho	UUO mice	[167]
Rhubarb	Emodin	BMP-7	Adriamycin-induced renal fibrosis rats	[168]
Rhubarb	Emodin	p38/MAPK pathway	Rat mesangial cells cultured under high glucose	[169]
Rhubarb	Emodin	TGF-β/Smad pathway	HK-2 cells	[170]
Rhubarb	Chrysophanol	TGF-β/Smad signaling pathway and the NKD2/NF-κB pathway	UUO mice	[171,172]

^a^NA: Not Applicable.

The difficulty in achieving large-scale standardized production mainly stems from the following reasons. Firstly, multiple factors including the place of origin, climate, picking time, and soil conditions can affect the components of TCM. Therefore, quality controls are essential for raw materials, such as morphological identification, DNA detection, and chemical component analysis [176]. Secondly, different processing methods of TCM may lead to different effects. Therefore, it is critical to explore the most suitable processing method and transform it into industrial production. Thirdly, the pharmacokinetic deficiency of TCM active ingredients may be the main reason for the rarity of clinical trials. Some methods for optimizing pharmacokinetics and enhancing bioavailability, including nanotechnology, micronized formulations, and combined preparation, have been proposed and validated in animal experiments and small-scale human trials [177]. Nevertheless, large-sample clinical trials are still necessary.

## 3. Precision medicine

Precision medicine refers to making medical adjustments based on the individual characteristics of each patient. It does not mean creating unique drugs or medical devices for patients, but the ability to divide individuals into different subgroups that differ in terms of susceptibility to specific diseases, the biology and/or prognosis of potentially developing diseases, or responses to specific treatments [178]. Many technologies, including artificial intelligence (AI), big data, organoids, and omics, are involved in precision medicine.

AI can provide potential targets and drugs for disease treatment more effectively and significantly reduce the time and cost of new drug development [179]. For instance, the small molecule TNIK inhibitor INS018_055 was discovered by the AI-driven drug discovery pipeline, and it has completed the Phase I clinical trial for idiopathic pulmonary fibrosis [180]. Similarly, INS018_073, a preclinical candidate drug for kidney fibrosis, was also discovered by the AI platform [181].

Renal organoids are gradually differentiated from embryonic stem cells (hESCs) or induced pluripotent stem cells (iPSCs) under the regulation of specific growth factors and ECM [182]. Specific and accurate renal fibrosis models can be made by the organoids technology to assist the mechanism research and drug development. The TGF-β-stimulating 3D renal fibrosis models were constructed which could reproduce the environment of renal fibrosis [183]. The hemolysis-induced kidney injury model was instructed by treating kidney organoids with hemin, and the anti-fibrotic efficacy of 4-(phenylthio) butanoic acid was verified in the model [184]. Additionally, organoids may be a method to solve the kidney shortage for transplantation. However, there are still many limitations, for example, the lack of vascularization and urinary function, heterogeneity, and safety issues [182].

Omics, including genomics, transcriptomics, proteomics, metabolomics, and epigenomics, focus on the function of biomolecules in diseases to clearly reveal pathogenesis and provide therapeutic targets. Xian Liao *etc*. discovered the ECM primary source was the PDGFRα+/PDGFRβ+ mesenchymal cells and revealed cell states of fibroblasts and myofibroblasts in human renal fibrosis [185]. Furthermore, the human epigenomic landscape of renal fibrosis has gained a new perspective, and some transcription factors have been found to potentially serve as targets for preventing CKD progression [186].

Additionally, precise medicine is helpful to reduce off-target effects of drugs. It is well known that pathways related to renal fibrosis also have other physiological functions, therefore, targeting these pathways may lead to off-target effects and potential toxicity. For example, TGFβ-1 plays an important role in growth and development. Embryonic mice with TGFβ-1 gene knockout mostly die before birth or shortly after birth [187,188]. TCM has multi-targets, which may also increase the probability of off-target effects. Tanshinone IIA inhibits angiogenesis through the PLC, Akt and JNK signaling pathways, thereby suppressing vascular endothelial growth factor and resulting in delayed tissue repair [189]. In the future, these problems may be solved through AI technology, drug-targeted delivery and preclinical trials of organoids.

However, the realization of precision medicine often relies on interdisciplinary cooperation and various cutting-edge technologies. Nevertheless, the high cost limits its development. Therefore, how to simplify technology, reduce costs, achieve industrialization and thereby realize universal benefits is the key issue to be addressed in the future.

## 4. Conclusion and perspectives

**Figure 4.**
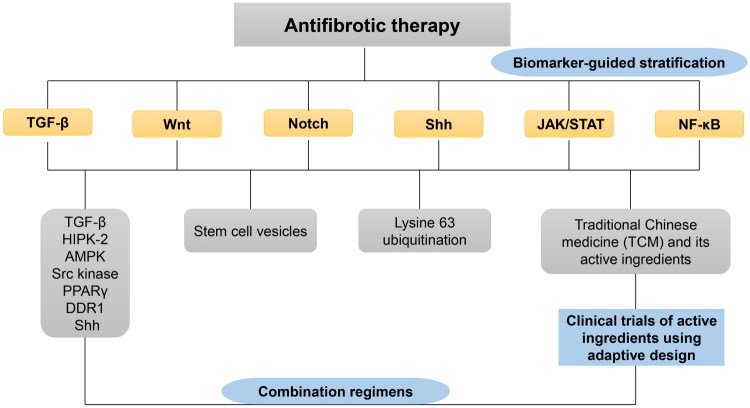
The roadmap of renal fibrosis treatment strategy.

Renal fibrosis is a chronic pathological process characterized by a complex network composed of various cytokines, inflammatory mediators, and signaling pathways (Figure 4). Understanding the interactions among these factors and exploring the core nodes are crucial for elucidating the underlying mechanisms. Firstly, extensive studies are still needed to identify specific biomarkers that indicate the initiation of renal fibrosis and guide stratification. Early diagnosis of renal fibrosis is essential for timely treatment. Employing cutting-edge biotechnological techniques, such as single-cell RNA sequencing for cell-type-specific fibrosis markers, utilizing big data to develop innovative algorithms, and integrating renal morphology, imaging, and hemodynamic assessments may enhance early diagnostic capabilities. Secondly, the mechanism of renal fibrosis is still unclear. Further research on the pathogenesis in renal fibrosis, especially exploring the potential reversibility of fibrosis with epigenetic reprogramming, may provide promising strategies for the complete treatment of renal fibrosis. Finally, effective treatment options for renal fibrosis remain a significant challenge. Although numerous drugs have been developed to target renal fibrosis, the clinical efficacy is limited. Combination regimens may provide better therapeutic effects by reducing the dosage of a single drug and alleviating the toxic side effects. Compared with Western medicine, the diverse active ingredients found in TCM have distinct advantages in modulating the networks of renal fibrosis. Therefore, identifying and purifying the effective components of TCM and synthesizing the corresponding compounds are becoming a hotspot in future research. Promoting the clinical transformation of these compounds will greatly improve the therapeutic effect of renal fibrosis. However, the components of TCM are diverse and the interactions among them are complex. Therefore, it is crucial to determine the active ingredients quickly and efficiently. Adaptive design means the experimental plan is dynamic and can be modified based on the real-time data obtained from the research, thereby improving research efficiency. In addition, using human kidney organoid models for drug screening is also an alternative option.

## Data Availability

The data of the analysis of keywords related to renal fibrosis and TCM in section 2.2 were derived from the PubMed database.
